# Obituary

**Published:** 1910-01-15

**Authors:** 


### Obituary.

Dr. William Clarke, M.D., has died at the age of eighty-three at Tankersley.

The death is reported of Dr. Julius Nicolaysen, well known in Norway as both physician and surgeon, who, at the centenary of the R.C.S. in 1900, was created an Honorary Fellow.

We have to announce the death of Mr. George Morton Wilson, late resident physician at Pendyffryn Hall Sanatorium, North Wales, at the age of forty-three. Mr. Wilson studied at Edinburgh University, where he gained the degrees of M.B. and C.M. in 1890.

Dr. John Holmes Joy, the Coroner for East Staffordshire, has died in his sixty-ninth year, at Tamworth, Staffordshire. Dr. Joy was a Dublin man, and became Master of Arts, Medicine, and Surgery at that University in 1865. The previous year he had gained his L.M. at the Rotunda Hospital. He was medical referee under the Workmen's Compensation Act, and district surgeon to the L.N.W. Railway.

Royal Society of Medicine.—The meeting of the Therapeutical and Pharmacological Section announced for January 18 will not take place. The next meeting of the Section will be held on Tuesday, February 1. At the next meeting of the Odontological Section, on Monday, January 24, at 8 p.m., the following papers will be read :—Dr. Eyre and Mr. J. Lewin Payne : "Some observations on the bacteriology of pyorrhœa alveolaris, and the results of treatment by bacterial vaccines." Mr. Payne will give his supplementary remarks. Mr. Kenneth Goadby : "The vaccine treatment of early cases of pyorrhœa alveolaris." The discussions on both papers will be taken simultaneously and opened by Mr. Goadby.

